# How are the youth? A brief‐longitudinal study on symptoms, alexithymia and expressive suppression among Italian adolescents during COVID‐19 pandemic

**DOI:** 10.1002/ijop.12866

**Published:** 2022-06-21

**Authors:** Cecilia Serena Pace, Guyonne Rogier, Stefania Muzi

**Affiliations:** ^1^ Department of Educational Sciences University of Genoa Genoa Italy

**Keywords:** COVID‐19, Internalizing and externalizing symptoms, Social media addiction, Binge eating, Expressive suppression, Alexithymia

## Abstract

Studies documented the negative consequences on adolescents' mental health of the stay‐at‐home measures adopted in reaction to the COVID‐19 outbreak. However, few contributions focused on the psychopathological trajectories after the end of these stressful measures or investigated the moderating role of this context in the relationship linking psychological symptoms with emotion regulation. This brief longitudinal study was performed with two measurement times: during the severe lockdown (T1), and when the restrictive measures were relaxed (T2). Ninety‐three community adolescents (45% boys; *M*_age_ = 14.94 years, *SD* = 1.64) completed the *Youth Self Report*, the *Social Media Disorder Scale*, the *Binge Eating Scale*, the *Emotion Regulation Questionnaire* and the *Toronto Alexithymia Scale 20 items*. Except for binge eating and externalising symptoms, all variables significantly decreased between T1 and T2. The relationship between expressive suppression and binge eating scores significantly decreased across time whereas the link between alexithymia and internalising symptoms increased with time. The study supported the idea that low‐risk adolescents experienced psychological relief from the relaxation of stay‐at‐home measures. Results suggest the importance of considering contextual factors when explaining the role of expressive suppression and alexithymia in binge eating and internalising symptoms among adolescents.

## Covid‐19 pandemic: long‐term impact of restrictive measures on adolescents' mental health

Since the last months of 2019, COVID‐19 has spread worldwide, constraining most national governments to adopt containment measures to limit its expansion and related consequences on the population's health. Therefore, from the beginning of 2020, several countries decided to implement stay‐at‐home measures, asking individuals to interrupt their usual job and social activities, going out only for necessity. In this context, also adolescents modified their daily life from several points of view: for example, from 10 March 2020, in Italy schools' lessons have been switched from face‐to‐face to digital, sports activities were interrupted and frequentation of peers was forbidden to teenagers. The restrictive measures may have been significant stressors for teenagers, as they resulted in teenagers' physical isolation, reducing the frequency and possibly the quality of their interpersonal and social interactions. Therefore, teenagers have been considered a vulnerable population, at risk of developing psychopathological symptoms due to the impact of lockdown measures (Muzi et al., 2021). Indeed, studies during the COVID‐19 pandemic highlighted that adolescents worldwide reported a diminution of emotional well‐being and heightened psychiatric symptoms (e.g., Hawke et al., 2020). Specifically, studies found that adolescents have shown increased levels of internalising and externalising symptoms (Cost et al., 2022), an internet (mis)use—including online gaming and addiction to social networks (Fernandes et al., 2021)—and an aggravation of symptoms in eating disordered adolescents (Serur et al., 2021).

Given a growing number of theoretical and empirical contributions stressed the human mind's capacity to recover (and even to grow) after experiencing a single highly stressful event (Tedeschi et al., 1998), at this stage a central issue is understanding the transitory (vs. permanent) nature of the impact of the COVID‐19 pandemic, lockdowns and consequences on adolescents' mental health, overcoming the exclusive documentation of short‐term adverse effects (Singh et al., 2020).

Recent longitudinal studies investigated the effects of the COVID‐19 pandemic on mental health collecting collected data in periods characterised by different restrictive measures. Some studies documented the persistence of depression and anxiety symptoms (Liu et al., 2021), and a worsening of alcohol intoxication emergencies among Italian youths after the strict lockdown's end (Grigoletto et al., 2020), overall suggesting that negative psychological impact of the outbreak persisted or worsened after the lockdown (Li et al., 2020). However, results in an extensive sample (van der Velden et al., 2021) showed that anxiety and depression levels measured after a relaxing of lockdown rules were lower than those in the pre‐pandemic period.

## Effects of the COVID‐19 outbreak on relationships between adolescents' mental health and emotion‐related risk factors

Before the COVID‐19 pandemic, specific adolescents' mental health problems were related to their individual differences in specific emotion‐related risk factors, such as *alexithymia* ‐defined as a deficit in emotional processing and awareness, including difficulties in identifying and describing somatic sensations and emotions, together with a proneness to show a concrete style of thinking—and *expressive suppression* (Gross & John, 2003)—defined as inhibition in expressing own emotional states, especially negative ones, without reducing the subjective and physiological experience of them—that is, traditionally considered as a maladaptive emotion regulation strategy. Indeed, pre‐pandemic studies showed that adolescents' internalising and/or externalising symptoms, social media misuses and binge‐eating symptoms were all predicted by high levels of alexithymia and/or expressive suppression (Garnefski et al., 2005; Ghamarani, 2012; Ling et al., 2012; Muzi & Pace, 2020; Muzi et al., 2021; Pace et al., 2021).

In line with these findings, specific adolescents' psychological problems during COVID‐19 pandemic may be related to their individual differences in alexithymia and expressive suppression, even if few studies investigated the relationships between these variables in the pandemic context. In a Chinese study, Zhang et al. (2021) observed that individuals (mostly adults) showing higher expressive suppression were more at risk of developing depression when psychologically distressed and exposed to social media. In contrast, Shao et al. (2021) seem to suggest a positive impact of expressive suppression during the pandemic, as they found that individuals who shared negative emotions (rather than keep them for themselves) showed a more negative reappraisal of pandemic‐related stressors. Finally, research conducted on a sample of Italian adults showed that an increase in emotional eating during the lockdown was associated with higher alexithymia scores (Cecchetto et al., 2021).

Together with findings of longitudinal effects of the pandemic on adolescents' mental health in the Italian or international context, also individual differences in alexithymia or expressive suppression can be affected by changes in lockdown restrictions, in line with a functional approach (Aldao & Nolen‐Hoeksema, 2012). Moreover, alexithymia or expressive suppression can, in turn, influence adolescents' capacity to recover from the lockdown's negative psychological impact over time. In this perspective, the exceptional life circumstances due to the pandemic—for example, almost 3 months of total lockdown, followed by gradual relaxing of restrictive measures—led to an unusual, potentially challenging social context, allowing to investigate whether the relationships between psychopathological outcomes, alexithymia and expressive suppression may vary as a function of contextual factors, specifically among adolescents.

As a whole, the knowledge of the long‐term reverberations of the COVID‐19 outbreak on adolescents' outcomes, both in terms of symptoms and emotion‐related variables, remains quite scarce and no studies investigated the extent by which the relationships between these variables change across contexts (during and after the lockdown period), calling for further investigation.

## The current study: research design and goals

**Figure 1:**
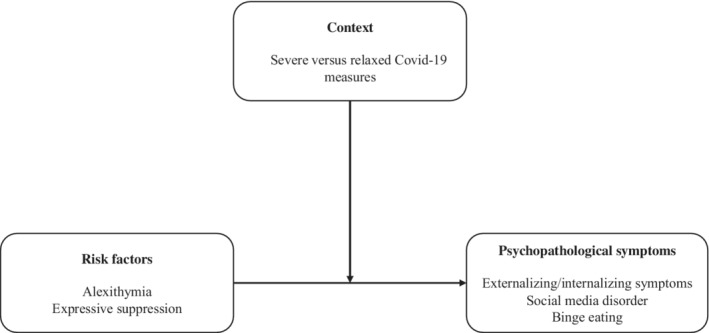
Conceptual moderation models tested.

To bridge the existing research gaps, a brief longitudinal study on Italian adolescents was conducted measuring a set of symptoms and emotion‐related risk‐factors in two steps: in April–May 2020, when the Italian government established severe restrictive measures, that is, stay‐at‐home and going out only for necessity (time 1, T1, from 10 April 2020 to 22 May 2020), and in September–October 2020 (time 2, T2, from 10 September 2020 to 19 October 2020), when restrictive measures were partially relaxed allowing to go out for non‐necessary motivations and students partly come back to schools in presence. Partial data collected only in T1 have been already published (Pace & Muzi et al., 2019). According to the framework surrounding the Post Traumatic Growth concept (Tedeschi et al., 1998), a reduction of symptoms would be expected from T1 to T2. Further, given that empirical and theoretical contributions stressed how contextual factors could impact individuals' alexithymia and expressive suppression, both emotion‐related risk factors were expected to change between T1 and T2. Indeed, alexithymia can be considered a stable personality trait or a state in response to stressors, and this study embraces this second perspective, especially because adolescents are building their personality and it appears inappropriate to assume stable personality traits. In the same vein, the propensity to use expressive suppression as a strategy of emotion regulation could vary across situations and life periods (Gross & John, 2003). Then, it was expected to observe significant correlations between symptoms variables and emotion‐related variables in both time 1 and 2. Moreover, it was expected to observe differences regarding these correlation patterns between these two sets of variables between T1 and T2. In other words, a moderation role of the time of measurement was expected with the role of these risk factors being stronger in the more stressful situation (T1) compared to T2. A graphical summary of the expected model is displayed in Figure 1.

## METHODS

### Participants and procedure

The sampling strategy aimed to recruit adolescents among the Italian community for larger longitudinal research. Therefore, directors of high schools were contacted to present the research through a presentation letter. If directors agreed to collaborate, MSc students in Psychology belonging to the research team illustrated the study to adolescents during online school lessons. Adolescents were briefly informed of the aims, scopes and procedure of the study and privacy guarantee. In case of availability to participate in the study, written informed consent was signed by adolescents and their parents, who received information about the study through a presentation letter attached.

The procedure complied with the ethical guidelines of the American Psychological Association and has been approved by the ethical board of the University of Genoa (Italy), protocol n. 037. After collecting the signed informed consent, in a first step (T1, from 10 April 2020 to 22 May 2020) MSc students used a research mail address to send an URL redirecting to an online survey to the adolescent's school email address. Having received a clinical diagnosis of a mental disorder was an exclusion criterion, to ensure a sample free from psychiatric diagnoses. The same link was sent 5 months after T1 (from 10 September 2020 to 19 October 2020, i.e., T2). On this online survey, the email contact of the principal investigator was provided in case the participant needed some additional explanations to fulfil the survey.

Of 125 Italian adolescents contacted through this sampling strategy, 22 adolescents (or their parents) refuse to participate, while 103 agreed to participate in this study and completed T1 data collection. Among these, 10 participants decided not to participate in T2. Therefore, the final group comprised 93 adolescents. High schools were all located in the urban area of the same city and *χ* ^2^ analysis did not evidence significant differences between schools in response rate (*p* = .224).

All participants attended secondary (27%) or high schools, belonging to six different schools; 45% of them were male, and their mean age was 14.94 years (*SD* = 1.64; Age range = 12–17 years). Computation of frequencies evidenced almost all adolescents reported medium economic level. Similarly, the sample was quite homogenous regarding parents' education levels, with 72% of adolescents' mothers and 66% of fathers having at least a high school degree. Lastly, 77% of adolescents reported their parents being married or living together, and 15% said their parents were divorced or separated. Multivariate analysis of variance evidenced a non‐significant omnibus effect (Pilliai's Trace = .05, *p* = .738) between adolescents participating at both T1 and T2 and adolescents dropping after T1 on the key variables of interest.

### Measures

The online survey included several self‐report questionnaires collecting data related to the following areas.

#### Internalising and externalising symptoms

Adolescents filled the widely used Youth Self Report (YSR; Achenbach, 2001), rating the occurrence of their symptoms in several syndromes sub‐scales, which return main scores of internalising problems (including symptoms of anxiety, withdrawal/depression and somatic complaints), externalising problems (including aggressive and delinquent behaviours) and a total score of problems, plus the main score of other problems not considered in this study. In this study, Cronbach's α reached a satisfying level being .88 and .80 for the internalising and externalising subscales respectively.

#### Social media disorder

The presence and levels of symptoms of addiction or problematic (mis)use of social media (e.g., Instagram, Facebook and online gaming) were measured with the Social Media Disorder Scale (SMDS; van den Eijnden et al., 2016; Marino et al., 2020), in a short version with nine yes/no questions. This study considered only the total score, which corresponds to higher levels of social media disorder as more as it increases. The version used in this study showed .76 Cronbach's α for reliability.

#### Binge eating

Prevalence and levels of binge eating symptoms were measured through the Binge Eating Scale (BES; Gormally et al., 1982; Ricca et al., 2000), a 16‐items questionnaire asking a respondent to choose between three/four different answers for each item, each one with scores 0–3. Scores range from 0 to 46, with a higher score indicating more severe binge eating symptomatology, and three cut‐off scores for prevalence: no binge eating with 17 or less, moderate with scores 18–26, and clinical levels of binge eating with 27 or more. Cronbach's α for the reliability of this version is .87.

#### Alexithymia

Alexithymia was self‐rated by adolescents through the widely‐validated questionnaire Toronto Alexithymia Scale 20‐items (TAS‐20; Bagby et al., 1994; Italian validation Bressi et al., 1996), which provides scores 1–5 in three factors Difficulty Identifying Feelings (DIF), Difficulty Describing Feelings (DDF) and Externally Oriented Thinking (EOT), also summed in a total score of alexithymia ranging 20–100. Higher scores correspond to higher levels of alexithymia, eventually indicative of individuals as not‐alexithymic if lower than 51, border‐alexithymic with scores 52–60 and alexithymic with a score of 61 or more. Cronbach's α for this version is .77.

#### Expressive suppression

This emotion regulation strategy was measured through the total score of expressive suppression in the Emotion Regulation Questionnaire for Children and Adolescents (ERQ‐CA; Gross & John, 2003; Italian validation Balzarotti et al., 2010), and the higher the score is, the more frequently the respondent use this strategy. The ERQ‐CA also provides a total score for the use of the Cognitive Reappraisal strategy, not considered in the current study. The reliability for the ES scale is .77.

### Statistical analyses

Statistical analyses were performed with the SPSS software v.23 and the Rstudio software for Mac. The first step of the analytic plan was preliminary, aiming to explore the internal consistencies of variables and to ensure the assumption of normality for the consequent test of hypotheses throughout parametric methods (see Table 1). Therefore, Cronbach's alphas were calculated for each of our variables, and QQ‐plots were visually inspected.

**TABLE 1:** Cronbach's alphas, skewness and kurtosis values for each variable involved in the study at step 1 and step 2

	*α*		Skewness		Kurtosis	
	Step 1	Step 2	Step 1	Step 2	Step 1	Step 2
YSR internalising	.88	.88	.52	.82	−.01	.39
YSR externalising	.80	.80	.88	.94	.48	.40
SMDS	.71	.88	.80	1.93	.14	3.13
BES	.83	.90	1.35	1.68	1.73	3.09
ERQ suppression	.70	.76	.08	.12	−.60	.25
TAS‐20 total	.76	.92	−.12	−.78	−.58	.26

*Note*: BES = Binge Eating Scale; ERQ = Emotion Regulation Questionnaire; SMDS = Social Media Disorder Scale; TAS‐20 = Toronto Alexithymia Scale 20 items; YSR = Youth Self Report.

Then, some descriptive analyses were performed by computing frequencies, means and standard deviations for demographic information and continuous variables. Also, to evaluate the need to insert age and gender as covariates in the subsequent analyses, some preliminary analyses were carried out. Specifically, Pearson's *r* correlations were computed to assess the relationship between age and our dependent variables, and multivariate analysis of variance, correcting the alpha inflation due to multiple comparisons with the Bonferroni method, was used to explore the presence of statistically significant differences between boys and girls on our continuous variables.

Multivariate mixed models were used to test the hypotheses that levels of all variables changed across time, and that time moderates the relationships between Independent Variables (i.e., TAS‐20; ERQ) and Dependent Variables (i.e., YSR, SMDS and BES). Also, Pearson's *r* correlation coefficient was used to explore links between continuous variables, separately in T1 and T2. Multivariate mixed models were performed using the bmrs package for R (Baldwin et al., 2014; Bürkner, 2017). The syntax used and the output are available in Appendix A. Complete correlations, variances and means are displayed in Appendix B.

## RESULTS

### Preliminary analyses and covariates check

Preliminary analyses showed that neither gender (*Pillai's Trace* = .16; *p* = .111) nor age (all *p*‐values >.234) were associated with any levels of continuous variables measured at T1, that is, YSR, SMDS, BES, TAS‐20 and ERQ scores. Consequently, gender and age were not used as covariates in the subsequent statistical analyses. In T2 there were no significant gender differences (*p* >.05), while age was positively and significantly correlated with YSR scores (*p* <.05).

### Symptoms and risk factors' changes from T1 to T2

The set of hypotheses of symptoms' change from T1 (severe stay‐at‐home restrictions) to T2 (relaxed restrictions) was tested. A significant decrease in total and internalising problems, and social media disorder symptoms from T1 to T2 was found while externalising problems and binge eating scores did not significantly differ between T1 and T2. Additionally, analyses showed that both total alexithymia and expressive suppression scores significantly decreased between T1 and T2. Results are displayed in Table 2.

**TABLE 2:** Multivariate mixed model testing changes of variables across time

	Means (SD)				
	Step 1	Step 2	ß	CI	R^2^
YSR total score	64.02 (21.53)	43.38 (28.93)	−11.68	−16.36 to −6.71	.53
YSR internalising	13.88 (8.50)	9.08 (8.51)	−3.12	−4.47 to −1.69	.72
YSR externalising	9.73 (6.51)	7.02 (6.50)	−2.89	0.02 to 1.00	.49
SMDS	2.52 (2.09)	1.29 (2.19)	−0.81	−1.37 to −0.24	.41
BES	6.53 (6.30)	6.45 (5.99)	−0.12	−1.56 to 1.26	.43
TAS‐20 total	53.47 (10.64)	37.58 (23.13)	−9.21	−13.64 to −4.76	.17
ERQ suppression	7.77 (3.36)	5.55 (4.18)	−1.21	−2.14 to −0.24	.30

*Note*: BES = Binge Eating Scale; ERQ = Emotion Regulation Questionnaire; SMDS = Social Media Disorder Scale; TAS‐20 = Toronto Alexithymia Scale 20 items; YSR = Youth Self Report.

### Changes across time in relationships between symptoms and risk factors

Given quite exploratory research's aims regarding changes over time in the relationships between symptoms and emotion‐related variables, bivariate correlations were first performed separately for T1 and T2. As Table 3 displays, results indicated that the TAS‐20 total score was significantly and positively correlated with YSR (total, internalising and externalising scores) and SMDS scores both at T1 and T2, and with BES scores only at T1. Moreover, ERQ‐CA_ES score was significantly and positively correlated with both SMDS and BES scores only at T1, and with YSR scores (total, internalising and externalising) only at T2.

**TABLE 3:** Bivariate correlations between all the variables involved in the study

	Time 1		Time 2	
	TAS‐20	Suppression	TAS‐20	Suppression
YSR total score	.41^**^	.17	.69^**^	.56^**^
YSR internalising	.43^**^	.20	.53^**^	.47^**^
YSR externalising	.33^**^	.15	.50^**^	.38^**^
SMDS	.30^**^	.28^**^	.35^**^	.11
BES	.28^**^	.35^**^	.19	.07

*Note*: BES = Binge Eating Scale; SMDS = Social Media Disorder Scale; TAS‐20 = Toronto Alexithymia Scale 20 items, Total score; YSR = Youth Self Report; *p <.05; **p <.01.

To test if these observed changes in the relationships between symptoms and emotion‐related risk factors could have been related to contextual variation in pandemic restrictions, a multivariate mixed model was performed to test the moderating role of time of measurement (i.e., T1 and T2) on the relationship between independent (TAS‐20 and ERQ‐CA_ES scores) and dependent (YSR, SMDS and BES scores) variables. Results (detailed in Table 4) revealed that Time significantly moderated the link between TAS‐20 total score and YSR_internalising as well as the relationship between ERQ‐CA_ES and BES scores. There were no other significant moderation effects.

**TABLE 4:** Multivariate mixed model testing the moderation role of time in the relationship between independent and dependent variables

	ß	SE	CI
*Intercept*			
YSR total score	31.61	18.70	−5.31 to 69.47
YSR internalising	−0.90	6.32	−13.49 to 10.95
YSR externalising	−4.62	5.91	−15.72 to 6.98
SMDS	−1.66	2.30	−6.07 to 3.10
BES	−7.22	5.92	−19.02 to 4.49
Time * TAS‐20			
YSR total score	−0.28	0.21	−0.69 to 0.12
YSR internalising	−0.16	0.07	−0.30 to −0.03
YSR externalising	−0.11	0.07	−0.24 to 0.02
SMDS	−0.00	0.03	−0.05 to 0.05
BES	−0.03	0.07	−0.17 to 0.10
Time * ES			
YSR total score	1.07	0.77	−0.44 to 2.50
YSR internalising	0.33	0.25	−0.15 to 0.81
YSR externalising	0.15	0.25	−0.34 to 0.62
SMDS	−0.16	0.09	−0.35 to 0.03
BES	−0.52	0.25	−0.99 to −0.03

*Note*: YSR: Youth Self Report; BES = Binge Eating Scale; ES = Expressive Suppression; *R* ^2^_BES_ = .49; *R* ^2^_SMDS_ = .47; *R* ^2^_YSR externalising_ = .59; *R* ^2^_YSR internalising_ = .64; *R* ^2^_YSR total_ = .75; SMDS = Social Media Disorder Scale; TAS‐20: Toronto Alexithymia Scale 20 items.

## DISCUSSION

The main aim of this study was to bring empirical evidence documenting the changes both in psychopathological symptoms (i.e., internalising and externalising, social media misuse, binge eating) and emotion‐related risk factors (alexythimia and expressive suppression) experienced by adolescents during diverse phases of the COVID‐19 pandemic, exploring changes in the relationships between risk factors and symptoms as well.

First, as expected, data showed that most of study variables significantly decreased between the first and the second measurement, suggesting that most of the adolescents' psychopathological manifestations were more marked in response to severe lockdown conditions, in line with previous studies (Fernandes et al., 2021; Cost et al., 2022; Hawke et al., 2020). Possible factors explaining these decreases are suggested by the literature (Pace & Muzi, 2019), for which the end of stay‐at‐home measures coincided with a reduction of stressors, resulting in a greater frequency of face‐to‐face interactions, a reduction of perceived loneliness, a decrease in teenagers' uncertainty about the future, and their perceived threat to their own and significant others' health, together with less frequency of ‐highly stressing‐ oral exams being typical of digital school. All these factors may have fostered the decrease of internalising and total problems, including anxiety and depression symptoms, as well as symptoms of social media disorder. Moreover, the latter result suggests teenagers' tendency to show social media misuse as transitory, emphasising the role of contextual stressors in influencing adolescents' symptoms. However, binge‐eating behaviours remained quite stable between the two phases of data collection, showing about 6% prevalence of binge‐eating risk, overlapping the one in previous studies with similar adolescents' samples (Pace et al., 2021). A similar result was found in relation to externalising symptoms. This suggests both binge‐eating behaviours and externalising symptoms as less sensitive to environmental changes, perhaps because maintenance factors for these disorders are early‐developed and they are usually particularly resistent (Fairburn et al., 2008). Regarding emotion‐related factors, this first set of results evidenced that both alexithymia and expressive suppression levels decreased from the first to the second measurement. This is not surprising in light of theoretical and empirical contributions converging towards the increase of emotion regulation efforts (both adaptive and maladaptive) in response to the frequency and intensity of negative emotional states (Sheppes et al., 2015; Velotti et al., 2021). Given that negative emotionality was probably high during the severe lockdown, it seems plausible that the end of this period led to the reduction in using (maladaptive) strategies to modulate negative emotions, such as alexithymia and expressive suppression.

Second, the hypothesis that changes in contextual factors (i.e., restrictions during or after the lockdown) would have moderated the relationship between emotion‐related factors and psychopathological symptoms was tested.

It emerged that adolescents with higher levels of alexithymia were more prone to experience externalising symptoms, as well as addiction to social media, both during severe restrictions (T1) and more relaxed ones (T2). Therefore, these results suggest alexithymia as a factor exercising a continuous impact on all these symptoms, regardless of environmental conditions. Notably, results showed a significant moderating role of Time in the relationship between alexithymia and internalising symptoms, which was stronger after the end of the severe lockdown period. This finding may be due to potential higher stress experienced by adolescents with internalising symptoms when turning to face‐to‐face interactions. Indeed, internalising symptoms include social anxiety and depression, which are psychopathological conditions often impacted by a perceived inability to interact adequately. Therefore, a possible explanation is that the negative impact of alexithymia may have been buffered during the lockdown, protecting adolescents from interpersonal triggers.

Regarding symptoms of addiction to social media, they were higher along with higher alexithymia both during and after the lockdown period. Again, this suggests that the difficulty in identifying and discriminating personal emotions accounts for compulsive use of social networks regardless of context variations. This result echoes the conclusions drawn from some data evidencing that alexithymia is a strong predictor of addiction to social media (Youssef et al., 2020). Theoretically, this would be explained by the proneness of an individual with high levels of alexithymia to use external regulators of adverse emotions instead of relying on more introspective and autonomous emotion regulation strategies requiring the capacity to identify and discriminate own internal states (Sheppes et al., 2015). Nevertheless, as suggested by other authors regarding internet addiction, interventions targeting emotional awareness in adolescents suffering from social media addiction appear promising (Rosenberg & Feder, 2014). Also, analyses evidenced that expressive suppression was associated with social media disorder only during the lockdown, but not after. Despite the moderation analyses not being significant, this result is particularly inspiring if interpreted in light of theoretical contributions asserting that addiction to social media may be explained by the individuals' difficulty expressing their emotional states freely. For instance, previous literature shed light on the fact that vulnerable narcissism, a pathological personality trait associated with the proneness to suppress the expression of negative emotional states, is a predictor of social media disorder (Casale & Fioravanti, 2018). Moreover, some authors argued that unauthenticity on social networks (i.e., camouflage of one's presentation) might serve as a gateway to developing or maintaining social media addiction (Gil‐Or et al., 2015). Therefore, in a lockdown condition where social media mediated most of the interactions with peers, adolescents more sensitive to the rewarding potential of social media activities may have been more vulnerable to a maladaptive use of social networks, while turning to face‐to‐face interactions may have weakened this risky process. This interpretation appears in line with the vicious circle theory between poor interpersonal skills and the rewarding value of social network consumption that would underline the development of social media addiction.

In addition, it was found that binge‐eating behaviours were associated with both alexithymia and expressive suppression during the lockdown but not after. Given levels of binge eating symptoms did not vary in the two measurement times, this result seems to suggest that context‐dependent variations may have impacted teenagers' likelihood to show binge‐eating behaviours in response to an increase in emotion‐related risk factors. In other words, this result, finding, further partially supported by results of moderation analysis testing the role of expressive suppression, is intriguing as, in the present study, binge‐eating behaviours levels did not vary across the two measurement times. Therefore, the role of context appears relevant to consider while evaluating the relationship between these two sets of variables. A potential explanation is that greater emotion regulation difficulties may have accounted for the development of dysfunctional eating habits during the lockdown, in line with theories for which individuals with binge eating cope with stressful events throughout the rewarding eating activity (Fairburn et al., 2008), and more interpersonally rooted mechanisms (e.g., social comparison) may have maintained these binge eating behaviours after the end of the lockdown. However, given there is no measure of binge eating prevalence before the lockdown in this group, future longitudinal studies are needed to test these relationships before, during, and after a stressful environmental change. Nevertheless, considering results brought by other studies documenting the worsening of both specific and general symptoms among individuals with eating disorders (e.g., Monteleone et al., 2021), our data may have relevant implications regarding the tailoring of interventions aimed to potentiate resilience against stressful triggers such as COVID‐19 related events. Also, the result may indicate that the use of expressive suppression is not maladaptive per se but that it depends on context, as elsewhere stated (Aldao & Nolen‐Hoeksema, 2012).

From a clinical perspective, our results suggest that targeting alexithymia in adolescents with internalising, externalising and social media disorder symptoms may be considered a transversal therapeutic strategy. Differently targeting the reduction of expressive suppression may be especially useful when adolescents with binge eating habits are experiencing an acute phase of stress.

A last intriguing finding concerns different patterns of correlations between age and internalising and externalising symptoms in the two steps. Indeed, while during severe restrictions (T1) no correlations were revealed between age and YSR scores, when the severe lockdown was relaxed, adolescents' age showed positive correlation with internalising and externalising symptoms, implicitly suggesting a stronger long‐term impact of lockdown in older adolescents than in younger ones, and this finding may be deepened with more sophisticated statistical analyses in future investigations.

### Limitations and future directions

This study brought inspiring results regarding the long‐term impacts of COVID‐19 on adolescents' mental health, but its conclusions should be considered in light of several limitations. First, there is no measure of the levels of psychopathological symptoms before the lockdown period, and this limits the capacity to interpret the observed changes between time 1 and time 2 as a recovery process. However, the plausibility of explaining the observed changes with the environmental variables (i.e., different lockdown conditions) found support in several studies documenting an increase of these psychopathological conditions among adolescents. This specific research design aimed to extend the current knowledge regarding the role of context in the relationship between emotion regulation and psychopathological symptoms that are currently essentially experimental. Future studies may want to replicate these results using an experimental procedure or more complex longitudinal studies, adding a measure of the subjective experience individuals make of the environmental factors. Then, the research illustrated in this contribution exclusively relied on self‐report instruments that may invalidate the results' validity. For instance, several authors shed light on the lack of reliability of the TAS‐20 among adolescents and on the utility of using an interview rather than a questionnaire to measure alexithymia features. Lastly, the study involved adolescents without a diagnosis of psychiatric disorder, limiting the generalisation of the results to clinical populations. Future studies may want to investigate further the topic, involving adolescents suffering from specific psychopathologies.

## Supporting information


**Appendix S1.** Syntax and output for international journal of psychology

**Appendix S2.** Correlations
