# Supplementary material for: How are the youth? A brief‐longitudinal study on symptoms, alexithymia and expressive suppression among Italian adolescents during COVID‐19 pandemic
Source: Int J Psychol. 2022 Jun 21:10.1002/ijop.12866. Online ahead of print. doi: 10.1002/ijop.12866 (PMC9350129; doi:10.1002/ijop.12866)
Supplement: Supplementary file 1 — Appendix S1. Syntax and output for international journal of psychology [file IJOP-9999-0-s001.pdf]

Code

- Show All Code
- Hide All Code
-
- Download Rmd

SYNTAX AND OUTPUT FOR INTERNATIONAL JOURNAL OF PSYCHOLOGY

This is an R Markdown Notebook. When you execute code within the notebook, the results appear beneath the code.

Try executing this chunk by clicking the *Run* button within the chunk or by placing your cursor inside it and pressing *Cmd+Shift+Enter*.

```
# Testing significant differences between T1 and T2 in every variable involved in the study
# INTER: Internalizing subscale of the Youth Self Report; EXTER: Externalizing subscale of the Youth Self Report; TOTYSR: Total Score of the Youth Self Report; BES: Binge Eating Scale; SMDS: Social Media Disorder Scale
bform3 <- bf(mvbind(INTER, EXTER, TOTYSR, BES, SMDS, SUPP, TASFT) ~ Time  + (1|p|id)) + set_rescor(TRUE)
fit3 <- brm(bform3, data = dati, chain=2, cores = 6, iter = 2000)
```

```
Rows containing NAs were excluded from the model.Compiling Stan program...
Start sampling

starting worker pid=36201 on localhost:11040 at 17:24:49.077
starting worker pid=36215 on localhost:11040 at 17:24:49.315

SAMPLING FOR MODEL '2835fbaaed67e0240549facbce7a2619' NOW (CHAIN 1).
Chain 1: 
Chain 1: Gradient evaluation took 0.000572 seconds
Chain 1: 1000 transitions using 10 leapfrog steps per transition would take 5.72 seconds.
Chain 1: Adjust your expectations accordingly!
Chain 1: 
Chain 1: 
Chain 1: Iteration:    1 / 2000 [  0%]  (Warmup)

SAMPLING FOR MODEL '2835fbaaed67e0240549facbce7a2619' NOW (CHAIN 2).
Chain 2: 
Chain 2: Gradient evaluation took 0.000545 seconds
Chain 2: 1000 transitions using 10 leapfrog steps per transition would take 5.45 seconds.
Chain 2: Adjust your expectations accordingly!
Chain 2: 
Chain 2: 
```

```
Chain 2: Iteration:    1 / 2000 [  0%]  (Warmup)
Chain 1: Iteration:  200 / 2000 [ 10%]  (Warmup)
Chain 2: Iteration:  200 / 2000 [ 10%]  (Warmup)
Chain 1: Iteration:  400 / 2000 [ 20%]  (Warmup)
Chain 2: Iteration:  400 / 2000 [ 20%]  (Warmup)
Chain 1: Iteration:  600 / 2000 [ 30%]  (Warmup)
Chain 2: Iteration:  600 / 2000 [ 30%]  (Warmup)
Chain 1: Iteration:  800 / 2000 [ 40%]  (Warmup)
Chain 2: Iteration:  800 / 2000 [ 40%]  (Warmup)
Chain 1: Iteration: 1000 / 2000 [ 50%]  (Warmup)
Chain 1: Iteration: 1001 / 2000 [ 50%]  (Sampling)
Chain 2: Iteration: 1000 / 2000 [ 50%]  (Warmup)
Chain 2: Iteration: 1001 / 2000 [ 50%]  (Sampling)
Chain 1: Iteration: 1200 / 2000 [ 60%]  (Sampling)
Chain 2: Iteration: 1200 / 2000 [ 60%]  (Sampling)
Chain 1: Iteration: 1400 / 2000 [ 70%]  (Sampling)
Chain 2: Iteration: 1400 / 2000 [ 70%]  (Sampling)
Chain 1: Iteration: 1600 / 2000 [ 80%]  (Sampling)
Chain 2: Iteration: 1600 / 2000 [ 80%]  (Sampling)
Chain 1: Iteration: 1800 / 2000 [ 90%]  (Sampling)
Chain 2: Iteration: 1800 / 2000 [ 90%]  (Sampling)
Chain 1: Iteration: 2000 / 2000 [100%]  (Sampling)
Chain 1:
Chain 1:  Elapsed Time: 25.0387 seconds (Warm-up)
Chain 1:                14.4448 seconds (Sampling)
Chain 1:                39.4835 seconds (Total)
Chain 1:
Chain 2: Iteration: 2000 / 2000 [100%]  (Sampling)
Chain 2:
Chain 2:  Elapsed Time: 26.6779 seconds (Warm-up)
Chain 2:                14.3081 seconds (Sampling)
Chain 2:                40.986 seconds (Total)
Chain 2:

Bulk Effective Samples Size (ESS) is too low, indicating posterior means and medians may be unreliable.
Running the chains for more iterations may help. See
https://mc-stan.org/misc/warnings.html#bulk-essTail Effective Samples Size (ESS) is too low, indicating posterior
variances and tail quantiles may be unreliable.
Running the chains for more iterations may help. See
https://mc-stan.org/misc/warnings.html#tail-essWarning messages:
1: package ‘StanHeaders’ was built under R version 4.0.2
2: replacing previous import ‘lifecycle::last_warnings’ by ‘rlang::last_warnings’ when loading ‘tibble’
3: replacing previous import ‘lifecycle::last_warnings’ by ‘rlang::last_warnings’ when loading ‘pillar’
Warning messages:
1: package ‘StanHeaders’ was built under R version 4.0.2
2: replacing previous import ‘lifecycle::last_warnings’ by ‘rlang::last_warnings’ when loading ‘tibble’
3: replacing previous import ‘lifecycle::last_warnings’ by ‘rlang::last_warnings’ when loading ‘pillar’

summary(fit3)

 Family: MV(gaussian, gaussian, gaussian, gaussian, gaussian, gaussian, gaussian)
  Links: mu = identity; sigma = identity
         mu = identity; sigma = identity
         mu = identity; sigma = identity
         mu = identity; sigma = identity
         mu = identity; sigma = identity
```

mu = identity; sigma = identity
mu = identity; sigma = identity
Formula: INTER ~ Time + (1 | p | id)
EXTER ~ Time + (1 | p | id)
TOTYSR ~ Time + (1 | p | id)
BES ~ Time + (1 | p | id)
SMDS ~ Time + (1 | p | id)
SUPP ~ Time + (1 | p | id)
TASFT ~ Time + (1 | p | id)
Data: dati (Number of observations: 157)
Draws: 2 chains, each with iter = 2000; warmup = 1000; thin = 1;
total post-warmup draws = 2000

Group-Level Effects:
~id (Number of levels: 91)

	Estimate	Est.Error	l-95% CI	u-95% CI	Rhat	Bulk_ESS	Tail_ESS
sd(INTER_Intercept)	6.60	0.63	5.42	7.84	1.01	319	515
sd(EXTER_Intercept)	4.33	0.55	3.17	5.40	1.01	331	337
sd(TOTYSR_Intercept)	13.71	1.95	9.54	17.08	1.02	148	68
sd(BES_Intercept)	3.88	0.79	2.04	5.30	1.01	294	357
sd(SMDS_Intercept)	1.40	0.24	0.91	1.86	1.00	326	564
sd(SUPP_Intercept)	1.86	0.37	1.10	2.57	1.00	373	670
sd(TASFT_Intercept)	3.93	1.96	0.42	7.78	1.00	252	495
cor(INTER_Intercept,EXTER_Intercept)	0.08	0.16	-0.25	0.35	1.01	236	210
cor(INTER_Intercept,TOTYSR_Intercept)	0.72	0.07	0.55	0.84	1.00	522	734
cor(EXTER_Intercept,TOTYSR_Intercept)	0.61	0.11	0.33	0.77	1.01	178	132
cor(INTER_Intercept,BES_Intercept)	0.31	0.15	-0.00	0.60	1.00	473	957
cor(EXTER_Intercept,BES_Intercept)	0.26	0.19	-0.15	0.59	1.02	281	321
cor(TOTYSR_Intercept,BES_Intercept)	0.47	0.16	0.14	0.76	1.02	310	579
cor(INTER_Intercept,SMDS_Intercept)	0.17	0.16	-0.16	0.48	1.00	511	777
cor(EXTER_Intercept,SMDS_Intercept)	0.21	0.18	-0.16	0.54	1.00	354	464
cor(TOTYSR_Intercept,SMDS_Intercept)	0.21	0.18	-0.15	0.55	1.00	356	691
cor(BES_Intercept,SMDS_Intercept)	0.03	0.21	-0.38	0.47	1.01	346	449
cor(INTER_Intercept,SUPP_Intercept)	0.41	0.16	0.08	0.71	1.01	594	875
cor(EXTER_Intercept,SUPP_Intercept)	0.25	0.20	-0.16	0.61	1.01	436	788
cor(TOTYSR_Intercept,SUPP_Intercept)	0.30	0.19	-0.09	0.64	1.01	279	371
cor(BES_Intercept,SUPP_Intercept)	0.14	0.23	-0.36	0.53	1.01	375	656
cor(SMDS_Intercept,SUPP_Intercept)	0.15	0.22	-0.28	0.59	1.00	483	771
cor(INTER_Intercept,TASFT_Intercept)	0.37	0.27	-0.32	0.79	1.00	931	743
cor(EXTER_Intercept,TASFT_Intercept)	-0.03	0.28	-0.58	0.49	1.00	970	1358
cor(TOTYSR_Intercept,TASFT_Intercept)	0.18	0.28	-0.44	0.66	1.00	953	1295
cor(BES_Intercept,TASFT_Intercept)	0.08	0.31	-0.55	0.64	1.00	783	824
cor(SMDS_Intercept,TASFT_Intercept)	-0.03	0.31	-0.62	0.56	1.00	852	1168
cor(SUPP_Intercept,TASFT_Intercept)	0.13	0.28	-0.47	0.65	1.00	953	1125

Population-Level Effects:

	Estimate	Est.Error	l-95% CI	u-95% CI	Rhat	Bulk_ESS	Tail_ESS
INTER_Intercept	17.17	1.22	14.79	19.58	1.00	678	1198
EXTER_Intercept	11.45	1.26	8.97	13.95	1.00	789	1150
TOTYSR_Intercept	76.39	3.92	69.20	84.40	1.00	657	909
BES_Intercept	6.50	1.14	4.25	8.79	1.00	1760	1727
SMDS_Intercept	3.34	0.47	2.43	4.27	1.00	1453	1234
SUPP_Intercept	8.99	0.76	7.52	10.52	1.00	1756	1457
TASFT_Intercept	62.56	3.44	55.80	68.90	1.00	1433	1373
INTER_Time	-3.10	0.70	-4.50	-1.76	1.00	890	1229
EXTER_Time	-1.50	0.80	-3.06	0.03	1.00	813	1084

TOTYSR_Time	-11.64	2.52	-16.67	-6.98	1.00	652	767
BES_Time	-0.09	0.73	-1.58	1.34	1.00	2154	1646
SMDS_Time	-0.81	0.30	-1.40	-0.22	1.00	1675	1399
SUPP_Time	-1.21	0.50	-2.19	-0.25	1.00	1871	1167
TASFT_Time	-9.23	2.25	-13.42	-4.90	1.00	1391	1348

Family Specific Parameters:

	Estimate	Est.Error	l-95% CI	u-95% CI	Rhat	Bulk_ESS	Tail_ESS
sigma_INTER	4.20	0.40	3.51	5.06	1.01	317	244
sigma_EXTER	4.49	0.43	3.76	5.41	1.01	242	196
sigma_TOTYSR	14.50	1.40	12.27	17.80	1.01	181	90
sigma_BES	4.39	0.50	3.52	5.51	1.00	341	462
sigma_SMDS	1.81	0.16	1.53	2.14	1.00	374	700
sigma_SUPP	2.95	0.22	2.54	3.40	1.00	405	903
sigma_TASFT	13.83	0.93	12.08	15.63	1.00	559	1152

Residual Correlations:

	Estimate	Est.Error	l-95% CI	u-95% CI	Rhat	Bulk_ESS	Tail_ESS
rescor(INTER,EXTER)	0.59	0.08	0.40	0.74	1.01	210	203
rescor(INTER,TOTYSR)	0.78	0.05	0.68	0.87	1.01	237	227
rescor(EXTER,TOTYSR)	0.87	0.03	0.80	0.92	1.01	232	174
rescor(INTER,BES)	0.30	0.12	0.04	0.53	1.00	386	574
rescor(EXTER,BES)	0.10	0.12	-0.15	0.34	1.01	302	535
rescor(TOTYSR,BES)	0.22	0.12	-0.02	0.44	1.01	335	700
rescor(INTER,SMDS)	0.09	0.12	-0.14	0.30	1.00	464	1151
rescor(EXTER,SMDS)	0.27	0.11	0.04	0.48	1.00	470	708
rescor(TOTYSR,SMDS)	0.25	0.11	0.02	0.46	1.00	457	893
rescor(BES,SMDS)	0.05	0.12	-0.21	0.28	1.01	622	875
rescor(INTER,SUPP)	-0.01	0.11	-0.22	0.20	1.01	699	1356
rescor(EXTER,SUPP)	0.02	0.11	-0.18	0.23	1.00	690	1407
rescor(TOTYSR,SUPP)	0.13	0.10	-0.08	0.33	1.01	587	1145
rescor(BES,SUPP)	0.21	0.11	-0.02	0.41	1.01	486	917
rescor(SMDS,SUPP)	0.09	0.11	-0.12	0.30	1.00	663	1136
rescor(INTER,TASFT)	0.29	0.10	0.08	0.46	1.00	645	1114
rescor(EXTER,TASFT)	0.38	0.09	0.19	0.56	1.00	905	1130
rescor(TOTYSR,TASFT)	0.43	0.08	0.26	0.58	1.01	714	970
rescor(BES,TASFT)	0.15	0.10	-0.05	0.35	1.00	772	1352
rescor(SMDS,TASFT)	0.35	0.09	0.15	0.52	1.00	839	1367
rescor(SUPP,TASFT)	0.40	0.08	0.24	0.55	1.00	1344	1458

Draws were sampled using sampling(NUTS). For each parameter, Bulk_ESS and Tail_ESS are effective sample size measures, and Rhat is the potential scale reduction factor on split chains (at convergence, Rhat = 1).

rr bayes_R2(fit3)

	Estimate	Est.Error	Q2.5	Q97.5
R2INTER	0.7223528	0.04871467	0.60578866	0.7987400
R2EXTER	0.4953948	0.08312473	0.29148550	0.6269776
R2TOTYSR	0.5347816	0.07758941	0.35727055	0.6577921
R2BES	0.4308379	0.11802426	0.15225202	0.6158960
R2SMDS	0.4100044	0.09485062	0.18723281	0.5646826
R2SUPP	0.3075122	0.08451891	0.14205286	0.4716745
R2TASFT	0.1783234	0.07387894	0.05152358	0.3341049

```
# INTER: Internalizing subscale of the Youth Self Report; EXTER: Externalizing subscale of the Youth Self
Report; TOTYSR: Total Score of the Youth Self Report; BES: Binge Eating Scale; SMDS: Social Media Disorder
Scale
bform2 <- bf(mvbind(INTER, EXTER, TOTYSR, BES, SMDS) ~ Time + SUPP + Time:SUPP +  TASFT +
Time:TASFT + (1|p|id)) +   set_rescor(TRUE)
fit2 <- brm(bform2, data = dati, chain=2, cores = 6, iter = 2000)

Rows containing NAs were excluded from the model.Compiling Stan program...
Start sampling

starting worker pid=36027 on localhost:11040 at 17:17:09.310
starting worker pid=36041 on localhost:11040 at 17:17:09.540

SAMPLING FOR MODEL '39fbecd601132901805c5c5325ab4121' NOW (CHAIN 1).
Chain 1:
Chain 1: Gradient evaluation took 0.000509 seconds
Chain 1: 1000 transitions using 10 leapfrog steps per transition would take 5.09 seconds.
Chain 1: Adjust your expectations accordingly!
Chain 1:
Chain 1:
Chain 1: Iteration:    1 / 2000 [  0%]  (Warmup)

SAMPLING FOR MODEL '39fbecd601132901805c5c5325ab4121' NOW (CHAIN 2).
Chain 2:
Chain 2: Gradient evaluation took 0.000466 seconds
Chain 2: 1000 transitions using 10 leapfrog steps per transition would take 4.66 seconds.
Chain 2: Adjust your expectations accordingly!
Chain 2:
Chain 2:
Chain 2: Iteration:    1 / 2000 [  0%]  (Warmup)
Chain 2: Iteration:  200 / 2000 [ 10%]  (Warmup)
Chain 1: Iteration:  200 / 2000 [ 10%]  (Warmup)
Chain 2: Iteration:  400 / 2000 [ 20%]  (Warmup)
Chain 1: Iteration:  400 / 2000 [ 20%]  (Warmup)
Chain 2: Iteration:  600 / 2000 [ 30%]  (Warmup)
Chain 1: Iteration:  600 / 2000 [ 30%]  (Warmup)
Chain 2: Iteration:  800 / 2000 [ 40%]  (Warmup)
Chain 1: Iteration:  800 / 2000 [ 40%]  (Warmup)
Chain 2: Iteration: 1000 / 2000 [ 50%]  (Warmup)
Chain 2: Iteration: 1001 / 2000 [ 50%]  (Sampling)
Chain 1: Iteration: 1000 / 2000 [ 50%]  (Warmup)
Chain 1: Iteration: 1001 / 2000 [ 50%]  (Sampling)
Chain 2: Iteration: 1200 / 2000 [ 60%]  (Sampling)
Chain 1: Iteration: 1200 / 2000 [ 60%]  (Sampling)
Chain 2: Iteration: 1400 / 2000 [ 70%]  (Sampling)
Chain 1: Iteration: 1400 / 2000 [ 70%]  (Sampling)
Chain 2: Iteration: 1600 / 2000 [ 80%]  (Sampling)
Chain 1: Iteration: 1600 / 2000 [ 80%]  (Sampling)
Chain 2: Iteration: 1800 / 2000 [ 90%]  (Sampling)
Chain 1: Iteration: 1800 / 2000 [ 90%]  (Sampling)
Chain 2: Iteration: 2000 / 2000 [100%]  (Sampling)
Chain 2:
Chain 2:  Elapsed Time: 64.6604 seconds (Warm-up)
Chain 2:                46.3677 seconds (Sampling)
Chain 2:                111.028 seconds (Total)
Chain 2:
```

```
Chain 1: Iteration: 2000 / 2000 [100%]  (Sampling)
Chain 1: 
Chain 1:  Elapsed Time: 67.3721 seconds (Warm-up)
Chain 1:                46.4556 seconds (Sampling)
Chain 1:                113.828 seconds (Total)
Chain 1: 

Bulk Effective Samples Size (ESS) is too low, indicating posterior means and medians may be unreliable.
Running the chains for more iterations may help. See
https://mc-stan.org/misc/warnings.html#bulk-essTail Effective Samples Size (ESS) is too low, indicating posterior
variances and tail quantiles may be unreliable.
Running the chains for more iterations may help. See
https://mc-stan.org/misc/warnings.html#tail-essWarning messages:
Warning messages:
1: package 'StanHeaders' was built under R version 4.0.2 
1: package 'StanHeaders' was built under R version 4.0.2 
2: replacing previous import 'lifecycle::last_warnings' by 'rlang::last_warnings' when loading 'tibble' 
3: replacing previous import 'lifecycle::last_warnings' by 'rlang::last_warnings' when loading 'pillar' 
2: replacing previous import 'lifecycle::last_warnings' by 'rlang::last_warnings' when loading 'tibble' 
3: replacing previous import 'lifecycle::last_warnings' by 'rlang::last_warnings' when loading 'pillar'

summary(fit2)

 Family: MV(gaussian, gaussian, gaussian, gaussian, gaussian) 
  Links: mu = identity; sigma = identity
         mu = identity; sigma = identity
         mu = identity; sigma = identity
         mu = identity; sigma = identity
         mu = identity; sigma = identity 
Formula: INTER ~ Time + SUPP + Time:SUPP + TASFT + Time:TASFT + (1 | p | id) 
         EXTER ~ Time + SUPP + Time:SUPP + TASFT + Time:TASFT + (1 | p | id) 
         TOTYSR ~ Time + SUPP + Time:SUPP + TASFT + Time:TASFT + (1 | p | id) 
         BES ~ Time + SUPP + Time:SUPP + TASFT + Time:TASFT + (1 | p | id) 
         SMDS ~ Time + SUPP + Time:SUPP + TASFT + Time:TASFT + (1 | p | id) 
   Data: dati (Number of observations: 157) 
  Draws: 2 chains, each with iter = 2000; warmup = 1000; thin = 1;
         total post-warmup draws = 2000

Group-Level Effects: 
~id (Number of levels: 91) 
                                    Estimate Est.Error l-95% CI u-95% CI Rhat Bulk_ESS Tail_ESS
sd(INTER_Intercept)                     6.33      0.64     5.15     7.66 1.01      544      932
sd(EXTER_Intercept)                     4.51      0.57     3.33     5.62 1.00      424      485
sd(TOTYSR_Intercept)                   13.61      1.69    10.38    16.86 1.00      397      714
sd(BES_Intercept)                       3.47      0.92     1.30     4.97 1.02      172      160
sd(SMDS_Intercept)                      1.42      0.25     0.89     1.88 1.00      494      805
cor(INTER_Intercept,EXTER_Intercept)    0.05      0.15    -0.27     0.33 1.01      389      609
cor(INTER_Intercept,TOTYSR_Intercept)   0.68      0.08     0.50     0.83 1.00      673     1015
cor(EXTER_Intercept,TOTYSR_Intercept)   0.64      0.10     0.40     0.79 1.01      356      584
cor(INTER_Intercept,BES_Intercept)      0.21      0.19    -0.19     0.54 1.00      445      538
cor(EXTER_Intercept,BES_Intercept)      0.20      0.21    -0.21     0.59 1.01      450      631
cor(TOTYSR_Intercept,BES_Intercept)     0.45      0.19     0.04     0.79 1.01      362      441
cor(INTER_Intercept,SMDS_Intercept)     0.16      0.16    -0.17     0.46 1.00      681     1222
cor(EXTER_Intercept,SMDS_Intercept)     0.26      0.18    -0.12     0.57 1.01      531      970
cor(TOTYSR_Intercept,SMDS_Intercept)    0.26      0.17    -0.11     0.58 1.01      497      967
cor(BES_Intercept,SMDS_Intercept)      -0.05      0.24    -0.55     0.42 1.01      244      416
```

Population-Level Effects:

	Estimate	Est.Error	l-95% CI	u-95% CI	Rhat	Bulk_ESS	Tail_ESS
INTER_Intercept	-1.28	6.35	-14.03	10.70	1.01	425	1083
EXTER_Intercept	-4.85	5.99	-16.77	6.41	1.01	492	887
TOTYSR_Intercept	30.75	18.79	-6.28	65.66	1.01	404	656
BES_Intercept	-7.70	6.25	-20.20	4.25	1.00	631	907
SMDS_Intercept	-1.67	2.30	-6.06	2.89	1.00	768	1276
INTER_Time	3.97	3.38	-2.47	10.83	1.01	480	867
INTER_SUPP	-0.48	0.41	-1.28	0.35	1.00	575	996
INTER_TASFT	0.40	0.13	0.16	0.64	1.01	399	754
INTER_Time:SUPP	0.33	0.25	-0.15	0.81	1.00	562	1089
INTER_Time:TASFT	-0.16	0.07	-0.30	-0.03	1.01	432	663
EXTER_Time	4.10	3.24	-2.10	10.56	1.01	507	987
EXTER_SUPP	-0.25	0.40	-1.01	0.53	1.00	520	903
EXTER_TASFT	0.32	0.12	0.09	0.56	1.01	416	801
EXTER_Time:SUPP	0.15	0.25	-0.34	0.62	1.00	536	875
EXTER_Time:TASFT	-0.11	0.07	-0.24	0.02	1.01	431	786
TOTYSR_Time	-0.36	10.26	-19.84	20.05	1.01	421	796
TOTYSR_SUPP	-1.40	1.23	-3.70	1.07	1.00	450	782
TOTYSR_TASFT	0.97	0.38	0.23	1.72	1.01	353	635
TOTYSR_Time:SUPP	1.07	0.77	-0.44	2.50	1.00	453	718
TOTYSR_Time:TASFT	-0.28	0.21	-0.69	0.12	1.01	373	526
BES_Time	6.12	3.40	-0.23	12.98	1.00	723	1014
BES_SUPP	1.09	0.40	0.30	1.83	1.00	955	1235
BES_TASFT	0.10	0.12	-0.13	0.35	1.00	652	857
BES_Time:SUPP	-0.52	0.25	-0.99	-0.03	1.00	970	1379
BES_Time:TASFT	-0.03	0.07	-0.17	0.10	1.00	692	1032
SMDS_Time	0.98	1.27	-1.63	3.38	1.00	871	1112
SMDS_SUPP	0.23	0.15	-0.07	0.52	1.00	1126	1313
SMDS_TASFT	0.05	0.05	-0.03	0.14	1.00	743	1149
SMDS_Time:SUPP	-0.16	0.09	-0.35	0.03	1.00	1208	1213
SMDS_Time:TASFT	-0.00	0.03	-0.05	0.05	1.00	808	1069

Family Specific Parameters:

	Estimate	Est.Error	l-95% CI	u-95% CI	Rhat	Bulk_ESS	Tail_ESS
sigma_INTER	4.09	0.39	3.39	4.92	1.01	527	1005
sigma_EXTER	4.06	0.39	3.39	4.95	1.00	444	390
sigma_TOTYSR	12.98	1.18	10.97	15.52	1.00	416	468
sigma_BES	4.39	0.54	3.50	5.56	1.01	190	199
sigma_SMDS	1.67	0.16	1.39	2.02	1.00	566	917

Residual Correlations:

	Estimate	Est.Error	l-95% CI	u-95% CI	Rhat	Bulk_ESS	Tail_ESS
rescor(INTER,EXTER)	0.52	0.09	0.33	0.68	1.01	421	891
rescor(INTER,TOTYSR)	0.76	0.05	0.64	0.85	1.00	594	1097
rescor(EXTER,TOTYSR)	0.85	0.04	0.77	0.91	1.01	402	671
rescor(INTER,BES)	0.37	0.12	0.11	0.58	1.00	337	632
rescor(EXTER,BES)	0.10	0.13	-0.16	0.36	1.02	435	996
rescor(TOTYSR,BES)	0.23	0.12	-0.02	0.47	1.02	279	510
rescor(INTER,SMDS)	-0.03	0.12	-0.26	0.23	1.00	654	1203
rescor(EXTER,SMDS)	0.13	0.12	-0.12	0.36	1.00	654	993
rescor(TOTYSR,SMDS)	0.12	0.12	-0.12	0.35	1.00	573	954
rescor(BES,SMDS)	-0.01	0.13	-0.26	0.25	1.01	416	814

Draws were sampled using sampling(NUTS). For each parameter, Bulk_ESS

and Tail_ESS are effective sample size measures, and Rhat is the potential
scale reduction factor on split chains (at convergence, Rhat = 1).

rr bayes_R2(fit2)

```
         Estimate  Est.Error      Q2.5     Q97.5
R2INTER  0.7471683 0.04382968 0.6467628 0.8179383
R2EXTER  0.5949335 0.08052342 0.3995892 0.7119968
R2TOTYSR 0.6402776 0.06802752 0.4797937 0.7416898
R2BES    0.4659216 0.10711091 0.2117473 0.6372736
R2SMDS   0.4937720 0.09102429 0.2764276 0.6391372
```

Add a new chunk by clicking the *Insert Chunk* button on the toolbar or by pressing *Cmd+Option+I*.

When you save the notebook, an HTML file containing the code and output will be saved alongside it (click the *Preview* button or press *Cmd+Shift+K* to preview the HTML file).

The preview shows you a rendered HTML copy of the contents of the editor. Consequently, unlike *Knit*, *Preview* does not run any R code chunks. Instead, the output of the chunk when it was last run in the editor is displayed.
