# Supplementary material for: How are the youth? A brief‐longitudinal study on symptoms, alexithymia and expressive suppression among Italian adolescents during COVID‐19 pandemic
Source: Int J Psychol. 2022 Jun 21:10.1002/ijop.12866. Online ahead of print. doi: 10.1002/ijop.12866 (PMC9350129; doi:10.1002/ijop.12866)
Supplement: Supplementary file 2 — Appendix S2. Correlations [file IJOP-9999-0-s002.pdf]

Correlations

Notes

Output Created		06-MAY-2022 16:55:...
Comments		
Input	Active Dataset	DataSet1
	Filter	<none>
	Weight	<none>
	Split File	<none>
	N of Rows in Working Data File	200
Missing Value Handling	Definition of Missing	User-defined missing values are treated as missing.
	Cases Used	Statistics for each pair of variables are based on all the cases with valid data for that pair.
Syntax		CORRELATIONS /VARIABLES=INTER EXTER TOTYSR BES SMDS SUPP TASFT /PRINT=TWOTAIL SIG /STATISTICS DESCRIPTIVES /MISSING=PAIRWISE.
Resources	Processor Time	00:00:00,01
	Elapsed Time	00:00:00,00

[DataSet1]

Correlations

		INTER	EXTER	TOTYSR	BES	SMDS	SUPP
INTER	Pearson Correlation	1	,495	,827	,415	,301	,399
	Sig. (2-tailed)		,000	,000	,000	,000	,000
	N	190	190	190	164	189	179
EXTER	Pearson Correlation	,495	1	,807	,232	,386	,320
	Sig. (2-tailed)	,000		,000	,003	,000	,000
	N	190	190	190	164	189	179
TOTYSR	Pearson Correlation	,827	,807	1	,413	,409	,481
	Sig. (2-tailed)	,000	,000		,000	,000	,000
	N	190	190	190	164	189	179
BES	Pearson Correlation	,415	,232	,413	1	,048	,235
	Sig. (2-tailed)	,000	,003	,000		,543	,003
	N	164	164	164	166	166	162
SMDS	Pearson Correlation	,301	,386	,409	,048	1	,244
	Sig. (2-tailed)	,000	,000	,000	,543		,001
	N	189	189	189	166	192	181
SUPP	Pearson Correlation	,399	,320	,481	,235	,244	1
	Sig. (2-tailed)	,000	,000	,000	,003	,001	
	N	179	179	179	162	181	183
TASFT	Pearson Correlation	,512	,452	,656	,180	,388	,571
	Sig. (2-tailed)	,000	,000	,000	,022	,000	,000
	N	178	178	178	161	180	178

Correlations

		TASFT
INTER	Pearson Correlation	,512
	Sig. (2-tailed)	,000
	N	178
EXTER	Pearson Correlation	,452
	Sig. (2-tailed)	,000
	N	178
TOTYSR	Pearson Correlation	,656
	Sig. (2-tailed)	,000
	N	178
BES	Pearson Correlation	,180
	Sig. (2-tailed)	,022
	N	161
SMDS	Pearson Correlation	,388
	Sig. (2-tailed)	,000
	N	180
SUPP	Pearson Correlation	,571
	Sig. (2-tailed)	,000
	N	178
TASFT	Pearson Correlation	1
	Sig. (2-tailed)	
	N	181

Descriptives

Notes

Output Created		06-MAY-2022 16:57:...
Comments		
Input	Active Dataset	DataSet1
	Filter	<none>
	Weight	<none>
	Split File	<none>
	N of Rows in Working Data File	200
Missing Value Handling	Definition of Missing	User defined missing values are treated as missing.
	Cases Used	All non-missing data are used.
Syntax		DESCRIPTIVES VARIABLES=INTER EXTER TOTYSR BES SMDS SUPP TASFT /STATISTICS=MEAN VARIANCE.
Resources	Processor Time	00:00:00,00
	Elapsed Time	00:00:00,00

Descriptive Statistics

	N	Mean	Variance
INTER	190	11,36	77,799
EXTER	190	8,32	43,868
TOTYSR	190	53,27	761,721
BES	166	6,49	37,682
SMDS	192	1,88	4,975
SUPP	183	6,67	15,531
TASFT	181	45,44	387,058
Valid N (listwise)	157		
